# Analysis of research hotspots and trends in epididymitis from 2014 to 2025: a visual analysis based on bibliometrics and scientific graphs from multiple databases

**DOI:** 10.3389/fmed.2026.1748905

**Published:** 2026-04-20

**Authors:** XiaoFang Wang, ZhiPeng Jiang, Jing Li, Kaihua Tang, XiJian Luo, DeCan Liang, AnGuo Li, YongHua Zhang, YiChi Zhang, Jie Chen, ZhongHong Yuan, ZhouNuo Tan, ZhiBo Wang, Jie Xie, Lei Liu, Meng Liu, Wen Luo

**Affiliations:** Department of Urology, The Third Affiliated Hospital of Zunyi Medical University (The First People's Hospital of Zunyi), Zunyi, China

**Keywords:** bibliometrics, epididymitis, PubMed, trend analysis, VOSviewer, web of science

## Abstract

**Background:**

Epididymitis is a common urogenital infection in adult men caused by sexually transmitted pathogens, urinary tract infections, trauma, or autoimmune mechanisms, with etiologies varying by age. In addition to severe pain and reduced quality of life, it may result in complications such as abscess formation, testicular atrophy, and infertility. Despite its clinical significance, comprehensive analyses of research trends remain limited. Bibliometric and knowledge-mapping approaches can provide quantitative insights into the field’s development and hotspots.

**Methods:**

Using the Web of Science Core Collection and the PubMed Database, we retrieved literature related to epididymitis published from January 1, 2014, to September 10, 2025. Bibliometric analyses of publication volume, journals, authors, institutions, countries, and keywords were conducted using the Bibliometrix R package (v5.1.1), VOSviewer (v1.6.20), and CiteSpace (v6.4. R2). Scientific knowledge maps were generated to identify research hotspots and developmental trends in epididymitis.

**Results:**

A total of 497 publications from 2,887 authors across 955 institutions in 67 countries were analyzed. Annual output remained stable at 30–50 articles since 2014. Andrologia was the most influential journal, and Pilatz Adrian was the leading author. China produced the most publications, the United States had the highest total citations, and Germany showed the highest average citation impact and strongest international collaboration. Justus Liebig University Giessen ranked first among institutions. Research trends have shifted from clinical diagnosis toward pathogenic and immunological mechanisms. Human studies focus on clinical features and treatment outcomes, whereas animal studies emphasize immunoinflammatory mechanisms and reproductive impacts.

**Conclusion:**

Research on epididymitis is advancing from clinical observation toward molecular immunology and precision therapy. Future studies should further focus on sexually transmitted pathogens, the immunopathological mechanisms of epididymitis, and the relationship between chronic inflammation and male infertility.

## 1 Introduction

Epididymitis is a common infectious disease of the urogenital system in adult men, primarily presenting with epididymal swelling and pain, often accompanied by scrotal erythema and fever (1, 2). Although epididymitis can occur at any age, its incidence is highest among sexually active young and middle-aged men, in whom the condition is strongly associated with sexually transmitted pathogens such as *Neisseria gonorrhoeae* and *Chlamydia trachomatis* (2, 3). In contrast, in men over 35 years of age, the causative agents are predominantly uropathogenic *Escherichia coli* (3). In addition to bacterial infection, non-infectious factors such as trauma, urine reflux, and autoimmune responses may also induce aseptic epididymal inflammation (4). Ascending infection involving the testis may lead to epididymo-orchitis, whereas recurrent or chronic inflammation can cause obstruction of the spermatic ducts and impaired spermatogenesis, ultimately contributing to male infertility (5). Therefore, timely and effective diagnosis and treatment of epididymitis are crucial for safeguarding male reproductive health.

In recent years, multiple international guidelines and expert consensuses on the clinical management of epididymitis have been published (6, 7), and progress has been made in several areas, including disease mechanisms (5, 8, 9), antimicrobial therapeutic strategies (7), imaging-based diagnosis (10), and the management of chronic epididymal pain. Nonetheless, several gaps persist in the field. First, the etiological spectrum of epididymitis is complex and varies substantially among different age groups: younger men are primarily affected by sexually transmitted pathogens, whereas older men are more often affected by uropathogenic bacteria (3). Second, increasing attention has been directed toward the long-term consequences of epididymitis. Chronic inflammation may disrupt the blood–testis barrier, induce fibrosis and luminal obstruction of the epididymal ducts, and thereby increase the risk of male infertility (5, 11, 12). It has been reported that a considerable proportion of male infertility cases associated with reproductive tract infection or inflammation originate from chronic inflammatory conditions of accessory glands such as the epididymis or prostate (13). Finally, the pathological mechanisms underlying chronic epididymitis and epididymal pain remain incompletely understood, and standardized diagnostic and therapeutic protocols are still lacking.

Overall, current research remains fragmented and lacks a systematic, integrative perspective. Most studies focus on specific pathogens or isolated clinical issues, while comprehensive analyses summarizing the landscape of research in the field are limited. Therefore, this study employs bibliometric methods combined with scientific knowledge mapping to quantitatively analyze research on epididymitis from the past decade. The core questions addressed include: (1) Which authors, institutions, and countries have made the greatest contributions to epididymitis research? (2) What are the characteristics of the collaboration networks and academic influence in this field? (3) What are the thematic hotspots and evolutionary trends in epididymitis research? (4) What are the main clinical and preclinical research focuses for epididymitis?

## 2 Method

### 2.1 Data extraction

**Figure 1:**
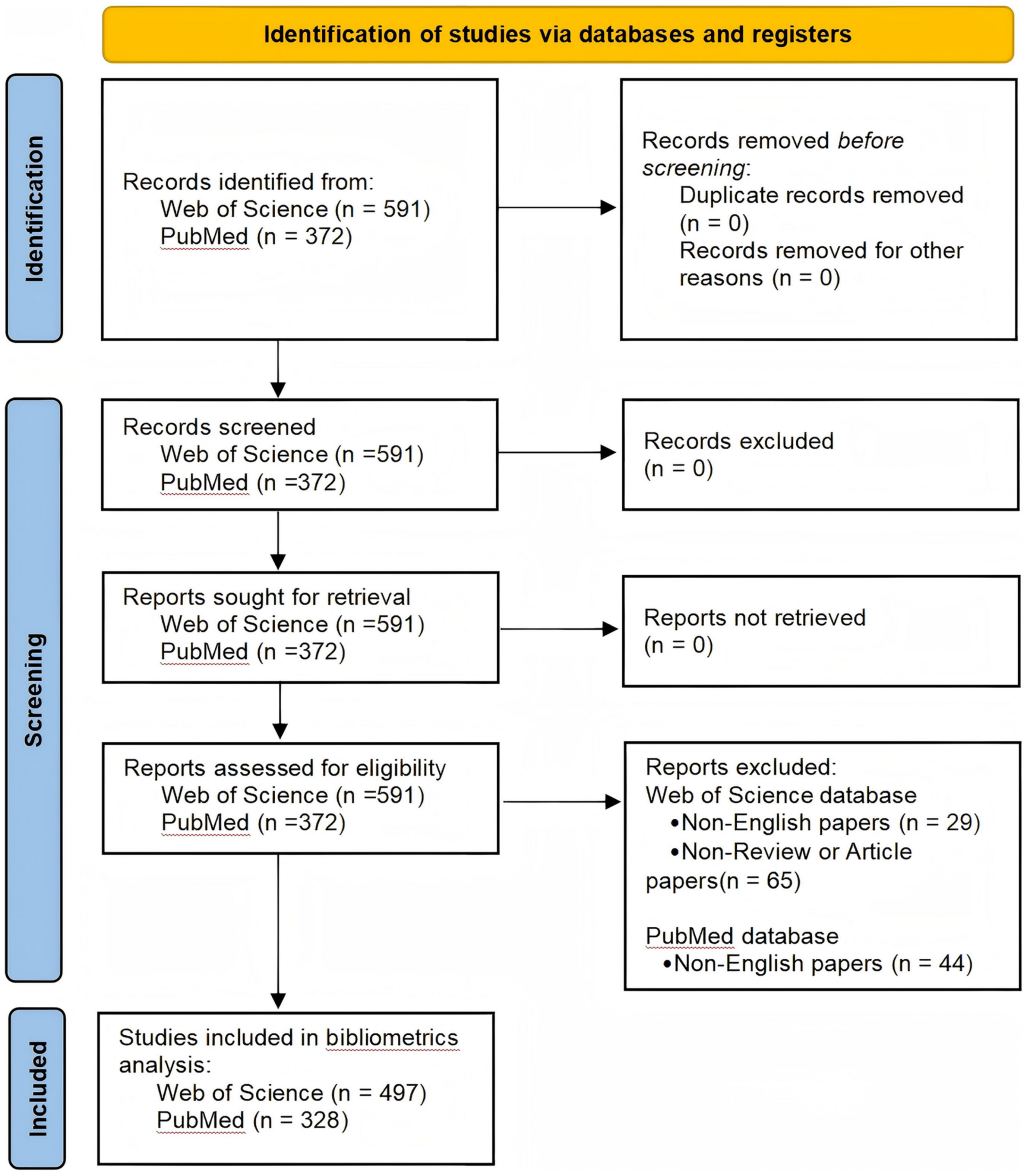
PRISMA 2020 flow diagram. Study identification and screening for epididymitis records retrieved from Web of Science Core Collection (SCI-E) and PubMed, showing numbers of records screened, excluded, and included for the bibliometric (WoS) and supplementary analyses (PubMed).

This study was conducted in accordance with the PRISMA 2020 data extraction and analysis guidelines (14). The data were obtained from the Web of Science Core Collection (Science Citation Index Expanded, SCI-E) and the PubMed database. Recognized as a highly authoritative database, the Web of Science Core Collection indexes high-quality academic journals and literature (15), making it the most suitable database for fundamental bibliometric analysis. In the preliminary search, we found that research on epididymitis has mainly focused on the past decade, covering various aspects, including molecular mechanisms, chronic inflammation and male infertility, pathogen spectrum characteristics, and antimicrobial treatment strategies. Based on this, to systematically reflect recent research progress in this field and enhance the timeliness and representativeness of the results, we limited the search time range to January 1, 2014, to September 10, 2025, with September 10, 2025, as the final search date of this study. This ensures the reproducibility of the research and minimizes the potential bias introduced by continuous database updates. A systematic search was conducted within this time frame, with inclusion criteria as follows: (1) the literature language was limited to English; (2) the document types were limited to Article or Review. After rigorous screening, a total of 497 articles meeting the criteria were identified (Table 1) and used as the data source for the bibliometric analysis in this study. To enhance the scientific quality and rigor of the research and to mitigate the limitations of relying on a single database, PubMed was used as a Supplementary data source. Relevant literature on epididymitis was searched (Table 1) and categorized into two groups based on species classification in PubMed: “Humans” and “Other Animals.” This categorization enabled a more objective understanding of trends and focus areas in both human and non-human research. The literature data from Web of Science and PubMed included above were initially screened for titles/abstracts and re-screened for full texts by two independent researchers, followed by data extraction and classification. To ensure data accuracy and research reproducibility, the two researchers compared their results, and any discrepancies were resolved through discussion with a third researcher. A detailed flowchart of the literature search and selection process is presented in Figure 1.

**Table 1:** Summary of data sources and selection criteria from the web of science core collection database and the PubMed database.

Category	Specific standard requirement	
Research database	Web of Science core collection(SCIE)	PubMed
Searching period	January 1, 2014–September 10, 2025	January 1, 2014–September 10, 2025
Language	English	English
Document or species types	Article or Review	Humans or Other Animals
Keywords	TS = (“epididymitis” OR “epididymitides” OR “epididymal inflammation” OR “inflammation of epididymis” OR “epididymal infection” OR “infection of epididymis” OR “acute epididymitis” OR “chronic epididymitis” OR “idiopathic epididymitis” OR “recurrent epididymitis”)	“Epididymitis”[Mesh]
Data extraction	Export with full records and cited references in plain	Export with all results in PubMed format
Initial sample size	591	372
Final sample size	497	328 (Humans: 278; other animals: 78)

### 2.2 Data analysis

Bibliometric analyses were performed using the Bibliometrix R package (v5.1.1), VOSviewer (v1.6.20), and CiteSpace (v6.4. R2). The analysis included annual publication volume, document types, journal distribution, author and institutional contributions, international collaboration, high-frequency keywords, collaboration networks among countries and institutions, author collaboration networks, and keyword co-occurrence networks. To achieve both breadth and depth in this study while enhancing scientific rigor, we leveraged the respective strengths of the two databases employed: Web of Science, with its broad multidisciplinary coverage, and PubMed, which offers high-quality biomedical literature. Specifically, in this study, data from PubMed were used to analyze trends in human and animal research, whereas the remaining bibliometric analyses were conducted using the Web of Science database.

### 2.3 Ethical statement

All data used in this study were obtained from publicly accessible publications and did not involve any human or animal experiments; therefore, no ethical approval was required. Data usage adhered to the service terms of the WOS and PubMed databases as well as the academic ethics guidelines.

## 3 Results

### 3.1 Basic information

**Figure 2:**
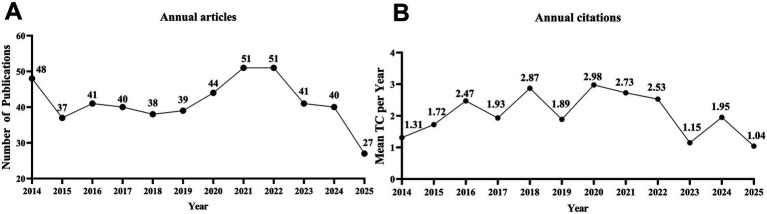
Annual publication output and citation impact. **(A)** Annual number of Publications. **(B)** Mean Total Citations per Year (MeanTC per Year).

A total of 497 publications were ultimately included in this study. From 2014 to 2024, the annual number of publications related to epididymitis exhibited a fluctuating trend (Figure 2). In 2014, 48 articles were published, after which the annual output generally remained between 30 and 50 publications per year. Regarding the mean number of citations per year (MeanTC per Year; Figure 2), citation frequency remained relatively low and stable throughout most of the study period. Notably, the mean annual citation rate began to rise after 2019.

### 3.2 Journal analysis

**Figure 3:**
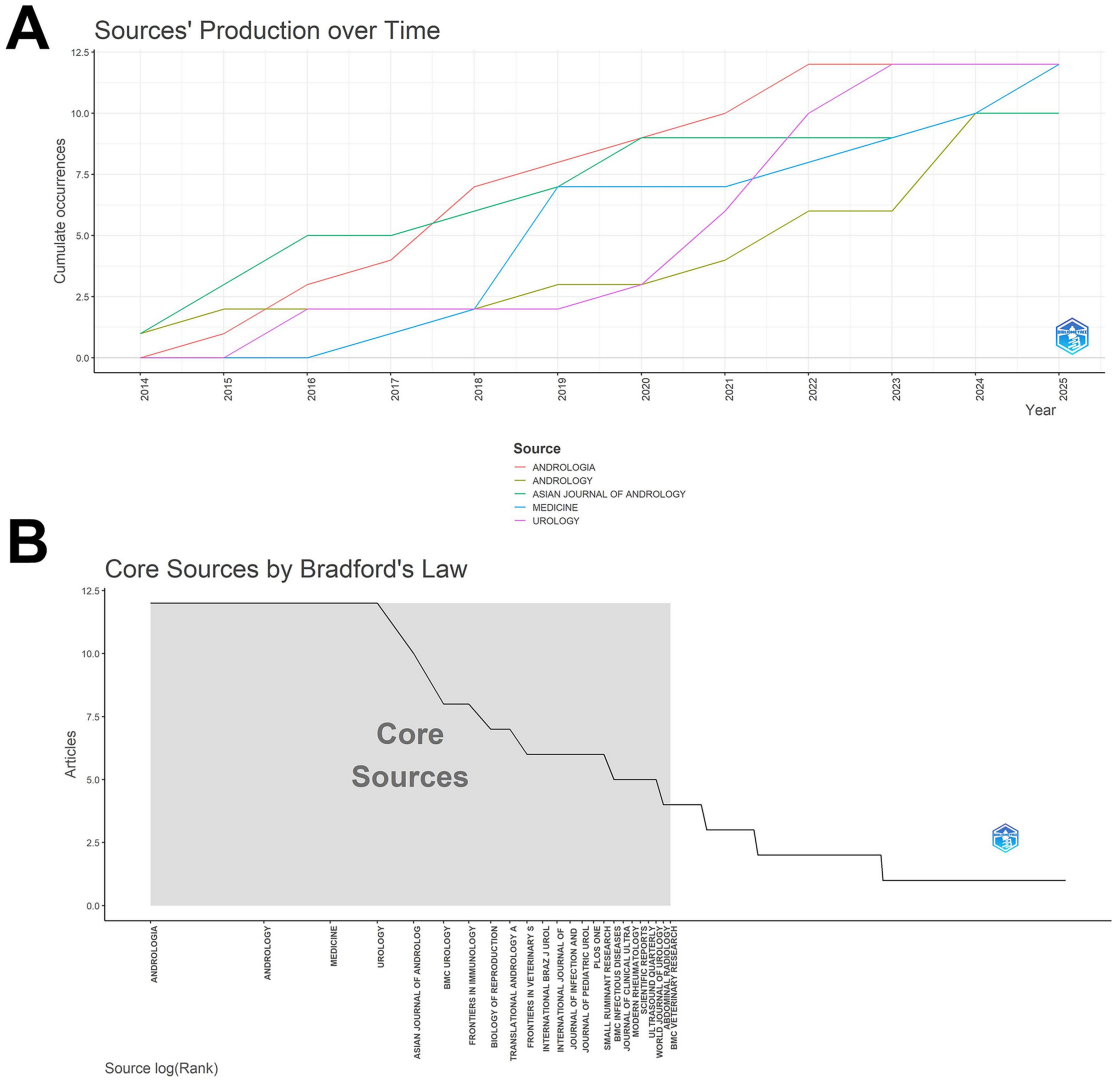
Journal analysis. **(A)** Source production over time for major journals, showing cumulative growth in field-specific outlets. **(B)** Bradford’s law distribution identifying core journals that contribute a disproportionate share of publications in this domain.

In this study, we observed a continuous upward trend in the publication output from major journals. Notably, journals such as *Andrologia* and *Andrology* showed significant increases in cumulative publication volume, reflecting growing research in epididymitis within these journals (Figure 3A). Core sources identified based on Bradford’s law of scattering (Figure 3B) revealed that journals such as *Andrologia*, *Andrology*, *Medicine*, and *Urology* constitute the core cluster, contributing the majority of high-quality literature. Among the top 10 most influential journals (Table 2), *Andrologia* ranked first with an h-index of 10, a g-index of 12, and a total citation count of 230. Other journals, such as *Asian Journal of Andrology* and *Biology of Reproduction*, also demonstrated high impact, underscoring their pivotal role in advancing epididymitis research.

**Table 2:** Top 10 journals impact by H index.

Rank	Journals	h_index	g_index	Total citation	Number of publications
1	Andrologia	10	12	230	12
2	Asian Journal of Andrology	8	10	196	10
3	Biology of Reproduction	6	7	143	7
4	Frontiers in Immunology	6	8	190	8
5	Andrology	5	12	176	12
6	Journal of Pediatric Urology	5	6	63	6
7	BMC Urology	4	8	77	8
8	Frontiers in Veterinary Science	4	6	61	6
9	Human Reproduction	4	4	68	4
10	International Braz J Urol	4	6	44	6

### 3.3 Author analysis

**Figure 4:**
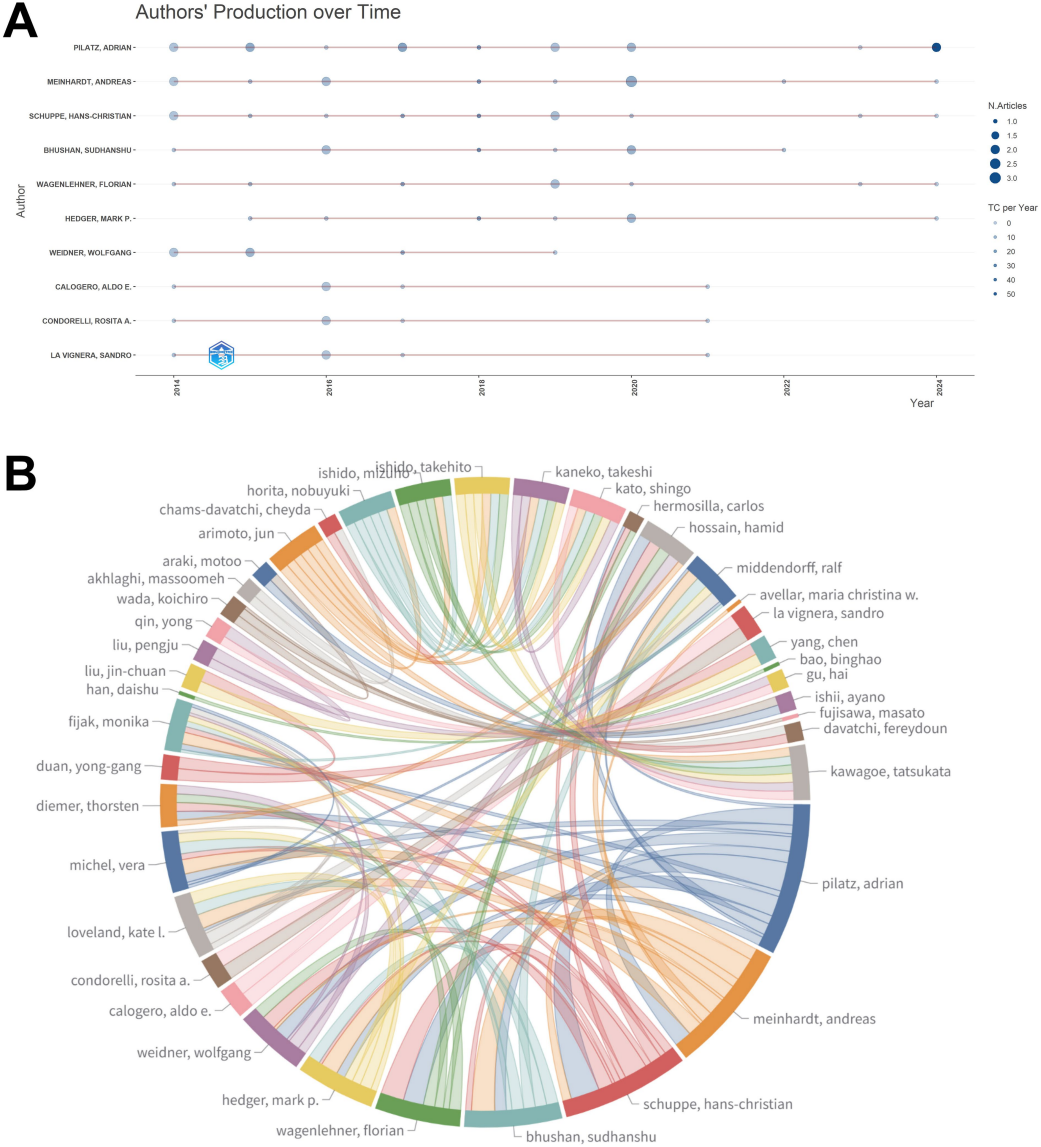
Author productivity and collaboration structure: **(A)** Authors’ production over time. Point size reflects annual publication volume and the overlay scale indicates citations per year. **(B)** Collaboration among authors. Chords represent co-authored links and chord width reflects collaboration strength, highlighting major collaborative hubs.

Among the authors analyzed, the most influential was Pilatz Adrian, who published 15 articles on epididymitis, with a total of 698 citations and an h-index of 11. The second most influential author was Meinardt Andreas, with an h-index of 10 and 12 publications (Table 3). The number of publications by different authors varied across different time periods (Figure 4A). The author collaboration network reveals a highly interconnected structure, with core nodes such as Pilatz Adrian, Meinardt Andreas, and Schuppe Hans-Christian, highlighting frequent cross-author collaborations (Figure 4B).

**Table 3:** Top 10 authors impact by H-index.

Rank	Author	h_index	g_index	Total citation	Number of publications
1	Pilatz Adrian	11	15	698	15
2	Meinhardt Andreas	10	12	437	12
3	Bhushan Sudhanshu	8	8	344	8
4	Schuppe Hans-Christian	8	11	480	11
5	P. Hedger Mark	6	7	355	7
6	Wagenlehner Florian	6	8	289	8
7	Weidner Wolfgang	6	6	284	6
8	L. Loveland Kate	5	5	126	5
9	Michel Vera	5	5	321	5
10	E. Calogero Aldo	4	5	46	5

### 3.4 Analysis of countries and institutions

In terms of countries and affiliations, the top 10 contributors by publication volume are listed in Tables 4, 5. The results indicate that China had the highest number of publications (110, 22.13%), followed by the United States (93, 18.71%) and Japan (31, 6.24%). The United States achieved the highest total citation count (1,331 citations, 14.3 citations per paper on average). Although Germany produced fewer papers (24), it exhibited the highest average citation rate (36.2 per paper), demonstrating strong research influence.

**Table 4:** Top 10 countries by the number of publications.

Rank	Country	Articles	Total citations	Average article citations	Articles %	SCP	MCP	MCP %
1	China	110	1,209	11	22.13	104	6	5.45
2	USA	93	1,331	14.3	18.71	86	7	7.53
3	Japan	31	341	11	6.24	28	3	9.68
4	Brazil	24	211	8.8	4.83	21	3	12.50
5	Germany	24	868	36.2	4.83	13	11	45.83
6	Italy	22	276	12.5	4.43	19	3	13.64
7	Turkey	17	174	10.2	3.42	15	2	11.76
8	India	14	180	12.9	2.82	9	5	35.71
9	Australia	12	231	19.2	2.41	9	3	25.00
10	Canada	12	159	13.2	2.41	9	3	25.00

CP, single-country publications; MCP, multi-country collaborations.

**Table 5:** Top 10 affiliations by the number of publications.

Rank	Affiliation	Articles
1	Justus Liebig Univ Giessen	18
2	Huazhong Univ Sci & Technol	6
3	Univ Catania	6
4	Nanjing Med Univ	5
5	Chinese Acad Med Sci	4
6	Fujian Med Univ	4
7	Massachusetts Gen Hosp	4
8	Univ Fed Minas Gerais	4
9	Univ Tehran Med Sci	4
10	Univ Wyoming	4

**Figure 5:**
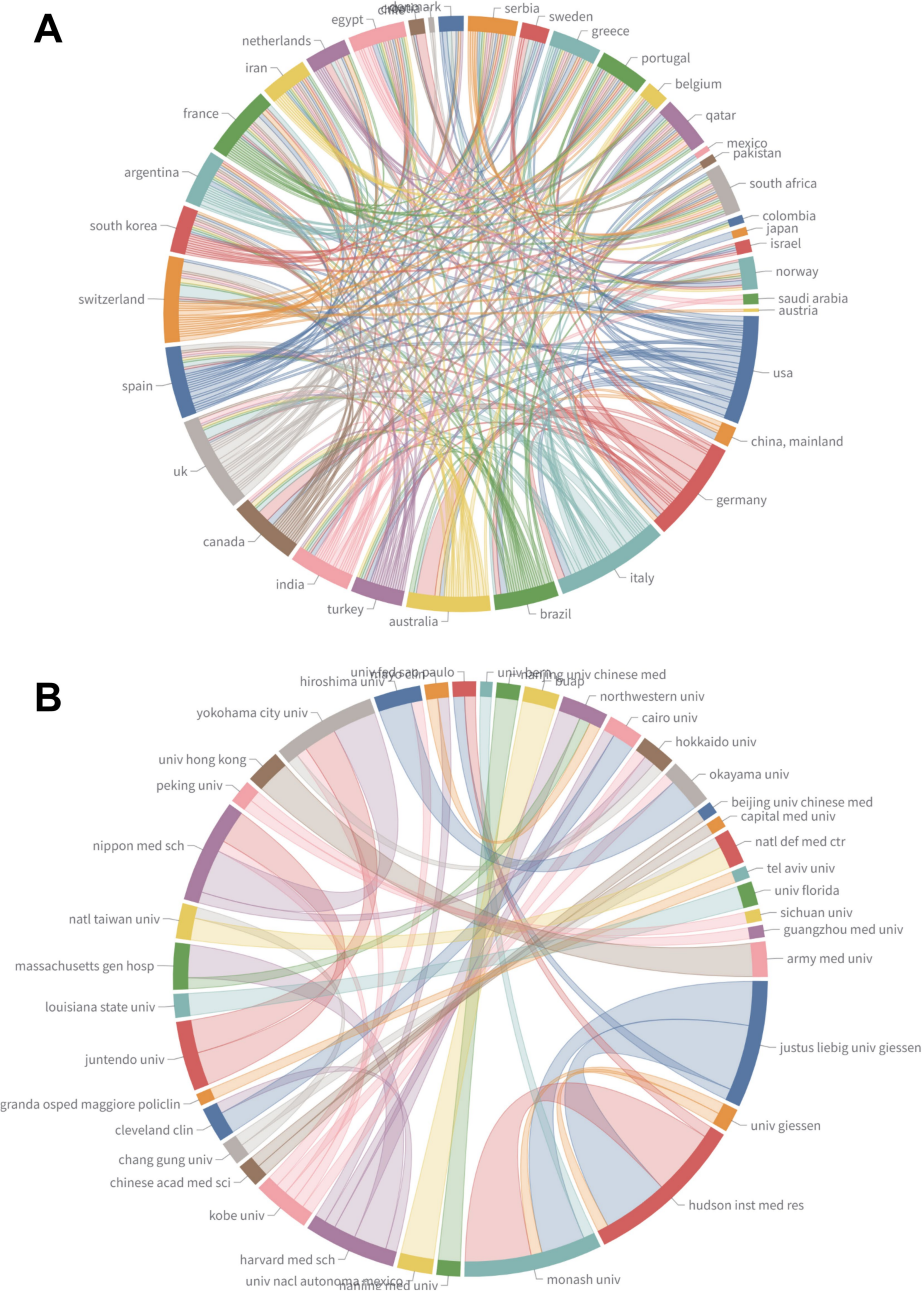
Collaboration networks among countries and institutions. **(A)** Country collaboration chord diagram; chord width indicates the intensity of international co-authorship and reflects single-country vs. multi-country collaboration patterns. **(B)** Institutional collaboration chord diagram showing a multi-centered, cross-regional cooperation structure; chord width represents inter-institutional collaboration strength.

Regarding international collaboration (Table 4), China showed a high proportion of single-country publications (SCP = 104) and a relatively low proportion of multi-country collaborations (MCP% = 5.45%). In contrast, Germany had the highest level of international cooperation (MCP% = 45.83%), consistent with the results of the international collaboration network (Figure 5A). The institutional collaboration network (Figure 5B) further revealed that research cooperation among institutions has evolved into a multi-centered, cross-regional pattern, reflecting increasing global integration in this field.

### 3.5 Keyword analysis

The three-field visualization analysis revealed that authors such as A. Meinhardt, A. Pilatz, and H. C. Schuppe are among the most prolific and highly cited researchers in this field. Their studies predominantly focus on keywords including “epididymitis,” “infection,” “inflammation,” and “infertility,” reflecting the central research themes surrounding the pathogenesis, infection pathways, and reproductive implications of epididymitis. This observation is further supported by the results of citation coupling–based literature clustering, which identified four distinct clusters: (1) an epididymitis research cluster centered on “epididymitis” and “inflammation”; (2) a complications cluster represented by “testicular torsion” and “infarction”; (3) a pathogenesis and infection mechanisms cluster associated with “bacterial epididymitis” and “epididymal tuberculosis”; and (4) a cluster focused on fundamental research into clinical manifestations such as “chronic pain” and “testicular pain.”

**Figure 6:**
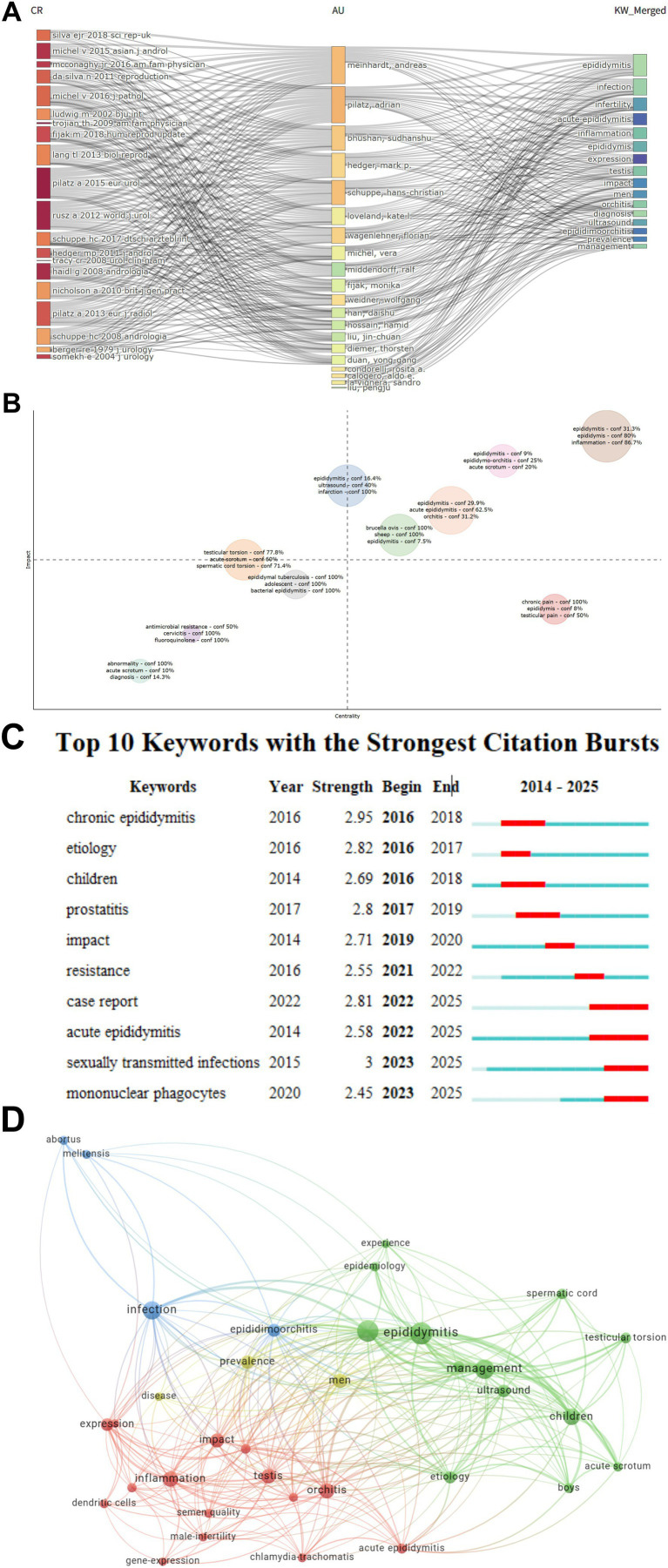
Knowledge structure, clustering, and keyword dynamics. **(A)** Three-field plot linking highly cited references, influential authors, and high-frequency keywords; band thickness denotes linkage strength. **(B)** Reference-coupling clustering of publications; distance reflects similarity in shared references and clusters capture major knowledge domains. **(C)** Top keywords with the strongest citation bursts; red segments indicate burst intervals and “Strength” denotes burst intensity. **(D)** Keyword co-occurrence network; node size indicates keyword frequency, link thickness reflects co-occurrence strength, and colors denote thematic modules: disease management and imaging-based diagnosis (green), infection and epidemiological characteristics (blue), inflammation and gene-expression mechanisms (red), and population-specific features with acute presentations (yellow).

The keyword co-occurrence network analysis further highlighted “epididymitis” as the central node, surrounded by high-frequency terms such as “management,” “infection,” “inflammation,” and “testis,” forming an intricate research network. The green module primarily addressed disease management and imaging diagnosis, the blue module emphasized infection and epidemiological characteristics, the red module explored inflammation and gene expression mechanisms, and the yellow module focused on the characteristics of different populations and acute cases. Keyword burst analysis revealed the temporal evolution of research topics: between 2016 and 2020, keywords such as “chronic epididymitis,” “etiology,” and “prostatitis” showed strong bursts, suggesting a research emphasis on etiology and clinical characteristics. Since 2022, terms including “acute epididymitis,” “case report,” and “sexually transmitted infections” have gained prominence and continue through 2025, indicating a gradual shift toward the infection mechanisms and clinical management of acute epididymitis caused by sexually transmitted pathogens. Overall, the research focus on epididymitis has evolved from early clinical etiological studies to a multidimensional framework encompassing pathophysiological mechanisms, immune inflammation, and clinical interventions (see Figure 6).

### 3.6 Theme evolution analysis

**Figure 7:**
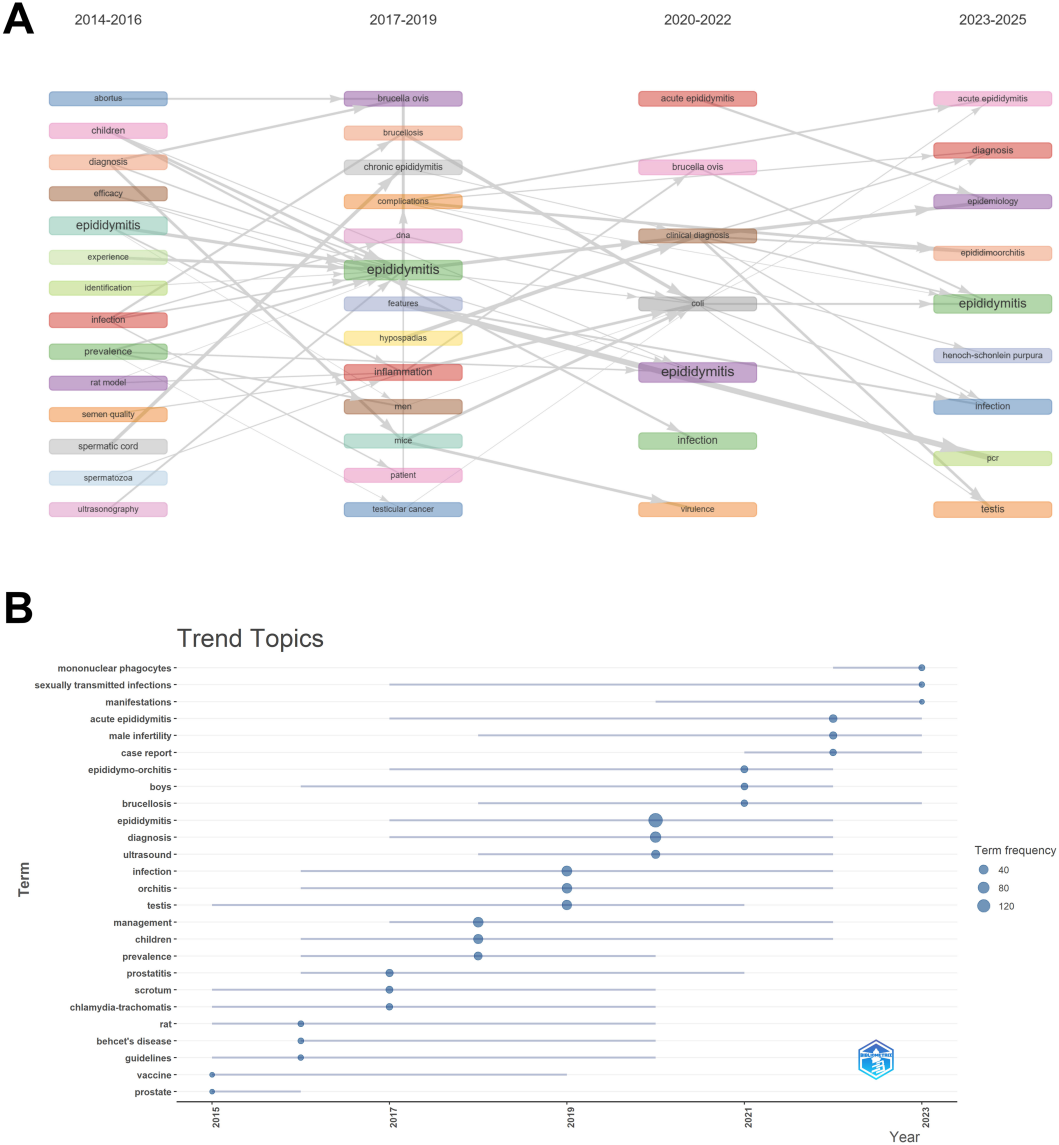
Thematic evolution and trend topics. **(A)** Alluvial map of main themes across four periods (2014–2016, 2017–2019, 2020–2022, 2023–2025); block size reflects prominence and flows indicate thematic continuity/shift over time. **(B)** Trend topic map showing temporal prominence of key terms; point size represents term frequency.

We conducted a visual analysis of the thematic evolution and research trends in epididymitis studies from 2014 to 2025 (Figures 7A,B). The results demonstrated a progressive shift in research focus over the past decade—from clinical diagnosis and epidemiology toward pathogen-related infection and immunological mechanisms. During 2014–2016, the dominant keywords included “epididymitis,” “infection,” “diagnosis,” and “children,” indicating that research during this period primarily addressed clinical manifestations, diagnostic methods, and disease characteristics in pediatric populations. Most studies at this stage were descriptive and focused on the clinical level. From 2017 to 2019, keywords such as “chronic epididymitis,” “inflammation,” “brucellosis,” and “complications” became more prominent, suggesting a growing focus on chronic inflammatory processes and pathogen-related features. This shift marks a transition from purely clinical observation to explorations of etiology and pathogenesis. During 2020–2022, research emphasis further evolved toward pathogen detection and animal models of acute epididymitis, while also expanding attention to infection routes and clinical classification. Between 2023 and 2025, the field has increasingly focused on epididymo-orchitis caused by sexually transmitted pathogens and on immune cell–mediated inflammatory responses. This trend indicates a deeper movement from clinical diagnosis and treatment toward investigations at the molecular and immunological levels. The thematic trend map (Figure 7B) further validates this evolutionary trajectory within the field.

### 3.7 Analysis of research progress in human and animal studies

PubMed is a high-quality medical database encompassing well-documented clinical trials and animal studies. Based on this advantage, we utilized PubMed to retrieve literature related to epididymitis and categorized the publications into human and animal research sections. This classification aims to provide researchers in the field with insights into current hotspots and trends in both human and animal studies.

**Figure 8:**
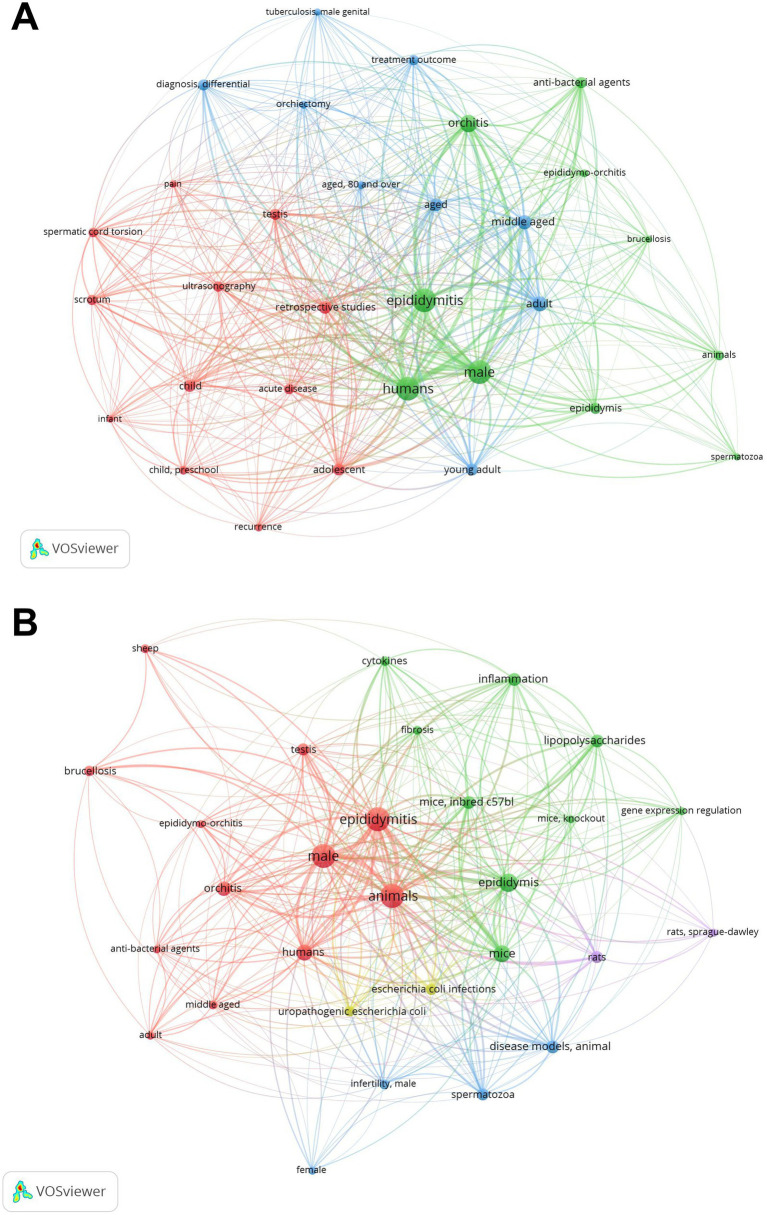
Keywords co-occurrence analysis graph in human and animal studies. **(A)** Human studies: network clustered into major clinical themes including epidemiology/etiology and age-related features, diagnosis/differential diagnosis with ultrasonography, and treatment/outcome evaluation. **(B)** Animal studies: network highlights experimental models, immune-inflammatory mechanisms of pathogen-induced epididymitis, and reproductive consequences. Node size indicates term frequency and link thickness indicates co-occurrence strength; colors denote clusters.

In the keyword co-occurrence analysis map for human research (Figure 8A), the network is divided into three major thematic groups. The results indicate that clinical research on epididymitis primarily focuses on: (1) epidemiological characteristics, etiology, and differences in clinical manifestations across various age groups; (2) clinical diagnosis, differential diagnosis, and ultrasonographic techniques; and (3) antibiotic therapy, surgical treatment, and outcome evaluation following intervention. In the corresponding co-occurrence analysis map for animal research (Figure 8B), the keyword network is organized into three major clusters. Key areas of focus include: (1) animal models commonly used to simulate human epididymitis; (2) immune and inflammatory mechanisms underlying pathogen-induced epididymitis; (3) the impact of epididymitis on the reproductive system, such as the spermatogenic microenvironment, spermatogenesis, and sperm quality.

## 4 Discussion

### 4.1 Basic information analysis

Between 2014 and 2024, a total of 497 publications on epididymitis were produced, with annual output consistently fluctuating within a narrow range of 30–50 articles. This relatively stable yet non-expansive publication trajectory contrasts with the rapid growth observed in certain related fields, such as male reproductive disorders (16, 17) or public health events (18). For instance, research on male infertility has demonstrated sustained expansion over the past two decades, accompanied by the progressive establishment of international collaborative networks (16), while the field of prostatitis has similarly shown notable increases in research scale and thematic refinement (17). Although research on epididymitis has not experienced comparable growth, this pattern should not be interpreted as indicative of limited scientific value; rather, it may reflect disparities in research resource allocation and academic prioritization. At the national level, China leads in publication volume, whereas the United States holds a clear advantage in total citation counts. This divergence may be partially attributable to differences in research funding intensity and the capacity to generate high-impact scientific outputs (19). The United States’ long-standing and stable biomedical funding infrastructure—particularly sustained investment in basic and translational research—likely contributes to its elevated citation influence. Nevertheless, the relationship between research investment and academic impact is not strictly linear. In recent years, China has experienced rapid growth in scientific output, reflecting expanded funding and an enlarged research workforce; however, its proportion of international collaboration and average citation impact remain comparatively modest. This structural pattern of “high output but relatively limited international collaboration” may constrain the global dissemination efficiency of its research findings. Bibliometric analyses have demonstrated that internationally co-authored publications tend to achieve higher citation impact, as multinational teams can enhance academic visibility through resource integration and complementary expertise (20). Accordingly, the comparatively low rate of international collaboration in Chinese epididymitis research may extend beyond mere differences in collaboration models, potentially involving language barriers, the maturity of academic networks, and research evaluation frameworks (19). In terms of journal distribution, epididymitis research is predominantly concentrated within specialized journals in andrology and urology, suggesting that knowledge dissemination in this field remains largely confined to disciplinary subdomains. In contrast, male infertility research has increasingly expanded into multidisciplinary fields encompassing reproductive medicine, molecular biology, and public health (16). Overall, epididymitis research over the past decade has exhibited structural stability but limited expansion, with scientific output largely driven by a small number of countries and with substantial room for deeper international collaboration. Strategic enhancements in funding allocation, transnational cooperation mechanisms, and interdisciplinary integration may therefore facilitate meaningful improvements in the global academic impact of epididymitis research.

### 4.2 Analysis of theme evolution and research hotspots and progress

The thematic evolution analysis, based on the chronological distribution of keywords, revealed a gradual transition in epididymitis research from early emphases on clinical manifestations, etiology, and diagnostic approaches to deeper investigations of disease mechanisms. With advances in immunology, research has increasingly focused on infection pathways and immune cell–mediated inflammatory responses, indicating a shift from clinical diagnosis and treatment toward molecular and immunological exploration. In addition, keyword co-occurrence and clustering analyses demonstrated the aggregation of themes such as “chronic epididymitis,” “immune barrier,” “sexually transmitted pathogens,” and “infertility,” reflecting several major research hotspots:

#### 4.2.1 Pathological mechanisms and immune microenvironment of chronic epididymitis

The thematic evolution analysis of this study indicates that chronic epididymitis and its associated immunoinflammatory mechanisms have emerged as a central frontier in epididymitis research over the past decade. Clinically, chronic epididymitis is frequently characterized by recurrent or refractory scrotal discomfort with persistent symptoms. Imaging and laboratory examinations often lack specificity, rendering both diagnosis and management challenging (21, 22). More importantly, a subset of patients continue to experience symptoms despite receiving standard antimicrobial therapy (23, 24), leading to a recognized therapeutic bottleneck. This observation suggests that the pathological basis of chronic epididymitis may extend beyond persistent infection and involve immune-driven structural alterations. Accumulating basic research evidence indicates that chronic inflammation can induce irreversible fibrotic remodeling within epididymal tissue. In a model of uropathogenic *Escherichia coli* infection, Michel and colleagues demonstrated that epididymitis results in marked interstitial fibrosis and luminal architectural disruption, with fibrotic changes persisting even after pathogen clearance (25). Furthermore, Wang et al. (26)reported that during bacteria-induced epididymitis, macrophages may undergo transdifferentiation into myofibroblast-like phenotypes, leading to excessive collagen deposition and promoting local fibrotic progression. These findings imply that immune cells not only participate in inflammatory responses but may directly mediate structural tissue remodeling, thereby establishing a self-perpetuating “inflammation–fibrosis–persistent inflammation” cycle. This mechanistic framework provides a plausible explanation for the refractory nature of chronic epididymitis and suggests that future therapeutic strategies may need to integrate anti-inflammatory, antifibrotic, and immunomodulatory approaches rather than relying solely on antimicrobial treatment.

In this context, the spatial heterogeneity of the epididymal immune microenvironment has emerged as a research focus. As a critical organ for sperm maturation and storage, the epididymis exhibits highly specialized immune regulation. It must maintain immune tolerance toward autoantigenic sperm while simultaneously preserving the capacity to defend against ascending infections. A review by Zhao et al. (5) highlighted significant segmental differentiation in immune function along the epididymis: the caput displays a relatively tolerogenic profile enriched in regulatory T cells and specific macrophage subsets, whereas the cauda possesses greater pro-inflammatory potential to enhance pathogen clearance. Pleuger et al. (8) further demonstrated that under infectious stimulation, distinct epididymal segments exhibit differential cytokine expression patterns and immune cell recruitment profiles. Collectively, these findings suggest that the initiation and progression of epididymitis may be segment-specific, with the magnitude and phenotype of immune responses shaped by local microenvironmental cues. Notably, chronic inflammatory states may also induce structural reorganization of local immune architecture. In a recent murine study, Elizagaray et al. (9) reported that sustained inflammation drives the formation of tertiary lymphoid structures within the epididymis, which are closely associated with autoimmune-related fertility impairment. This discovery implies that chronic epididymal inflammation may not only alter immune cell composition but also remodel local immune organization, potentially exerting long-term effects on reproductive function. Should similar immune remodeling be confirmed in humans, its clinical implications would extend well beyond infection control alone. Despite the promising trajectory of research on epididymal immune cells, several challenges remain. First, limited access to human epididymal tissue constrains direct translational investigation, necessitating reliance on animal models and cautious extrapolation of findings. Second, the high cost of single-cell sequencing and spatial transcriptomics technologies currently restricts large-scale human studies. Nevertheless, with the rapid advancement of spatial omics and immuno-multiomics approaches, it is reasonable to anticipate that investigations of the epididymal immune microenvironment will become increasingly refined and systematic over the next decade.

#### 4.2.2 Infectious pathogens and sexually transmitted factors

Our analysis identified sexually transmitted infection (STI)–related epididymitis as another major research hotspot over the past decade. In young men, epididymitis is closely associated with sexually transmitted pathogens such as *Neisseria gonorrhoeae* and *Chlamydia trachomatis*. Bibliometric findings demonstrated a marked increase in the frequency of STI-related keywords beginning in 2022, reflecting growing scholarly attention to infection routes and pathogen-specific mechanisms. This emerging focus carries important implications for both clinical practice and public health policy. First, given that gonorrhea and chlamydia represent the principal etiologies of epididymitis in younger patients, standardized STI prevention and control strategies are critical for reducing disease incidence. Multiple international guidelines emphasize timely screening and treatment of STIs to prevent complications (7, 27). For epididymitis caused by *N. gonorrhoeae* or *C. trachomatis*, authoritative guidelines recommend empirical therapy with a cephalosporin combined with doxycycline or a fluoroquinolone to ensure coverage of potential mixed infections (6, 7, 28). The endorsement of combination therapy reflects heightened awareness of increasing antimicrobial resistance and corresponding adjustments in treatment strategies in recent years. Second, advances in molecular diagnostics have substantially improved etiological identification in epididymitis. Conventional culture methods and routine nucleic acid testing fail to detect pathogens in approximately 20–30% of cases (29). In contrast, next-generation sequencing and multiplex PCR technologies have successfully identified causative organisms in previously unexplained cases. Pilatz and colleagues employed 16S rDNA sequencing to detect difficult-to-identify bacterial species in epididymitis, thereby refining prior understanding of its etiological spectrum (29). These findings suggest that future research will increasingly apply advanced molecular diagnostic approaches to delineate the full pathogen spectrum of epididymitis, including atypical organisms such as mycobacteria, parasites, and potentially viral agents. Emerging evidence has proposed the possibility of viral epididymitis (30); however, its epidemiology and pathogenesis remain insufficiently characterized and warrant further investigation. Such research will not only enhance diagnostic precision but also promote the continual refinement of treatment guidelines to enable pathogen-specific therapeutic strategies. Finally, the growing emphasis on STI-associated epididymitis underscores the need for strengthened public health initiatives. Enhanced sexual health education and targeted interventions aimed at reducing gonorrhea and chlamydia transmission may mitigate the burden of epididymitis at its source.

#### 4.2.3 Male infertility and reproductive health

Epididymitis has attracted increasing attention in relation to male fertility. Clinical and epidemiological evidence has long indicated that both acute epididymitis and recurrent chronic epididymitis can adversely affect spermatogenic and sperm storage functions of the testis and epididymis. Reported abnormalities include reduced sperm count, decreased motility, increased morphological defects, and altered seminal biochemical parameters (21, 31, 32). From a mechanistic perspective, the pathways linking epididymitis to infertility represent a major focus of current research. On the one hand, inflammation-induced tissue injury may directly disrupt the spermatogenic microenvironment. Chronic inflammation can trigger persistent immune activation, leading to focal impairment of spermatogenesis. Infiltrating inflammatory cells release reactive oxygen species and pro-inflammatory mediators that may compromise sperm DNA integrity and alter the seminal microenvironment (33). Histopathological studies have reported that a substantial proportion of patients with epididymitis exhibit chronic inflammatory changes on testicular biopsy, with patterns comparable to those observed in men with idiopathic infertility, suggesting a pivotal role for inflammation in unexplained male infertility (12, 13). On the other hand, epididymitis may exert subclinical effects on the molecular composition and functional capacity of sperm. Proteomic analysis by Pilatz et al. (34) demonstrated that even 3 months after clinical resolution of acute epididymitis, significant alterations persisted in the sperm protein expression profile, including the downregulation of 35 proteins associated with sperm function. These molecular perturbations may interfere with critical processes such as sperm maturation and capacitation, thereby constituting a latent contributor to post-epididymitis infertility. In addition, autoimmune responses triggered by chronic epididymitis (9, 35) have been proposed as another pathogenic mechanism. Sustained inflammation may disrupt epididymal–testicular immune tolerance, promote the generation of antisperm antibodies, or induce autoimmune-mediated tissue injury, ultimately impairing reproductive capacity. Given the multifaceted impact of epididymitis on male fertility, two major implications for clinical translation emerge. First, greater emphasis should be placed on screening for occult epididymitis in men with unexplained infertility. Routine infertility assessments often overlook prior epididymal inflammation or subclinical epididymal damage. Future strategies may incorporate evaluation of seminal leukocytes, inflammatory biomarkers, and targeted imaging to facilitate early detection. Second, adjunctive reproductive interventions targeting inflammation-related infertility warrant development. For men with persistent semen abnormalities following epididymitis, it remains to be determined whether anti-inflammatory or antioxidant therapies, or optimized semen processing techniques, can improve reproductive outcomes. In summary, the present findings underscore that epididymitis should not be regarded solely as an infectious condition but also as a significant risk factor for male infertility. This recognition calls for heightened attention in both research and clinical practice, thereby supporting the development of evidence-based management guidelines and fertility-preservation strategies.

## 5 Research limitations

This study has several limitations. First, data collection is subject to temporal lag and coverage constraints. Articles indexed in PubMed and Web of Science during the submission period, as well as newly published studies in this field that had not yet been indexed, were not included in the analysis. Second, the study was restricted to publications in English, which may have resulted in the omission of relevant literature and introduced a degree of selection bias. Nevertheless, given that Web of Science and PubMed are the two most authoritative and widely used international scientific databases, encompassing the majority of high-quality research, and considering the extended time span covered in this study, such potential bias is unlikely to materially affect the overall conclusions. In addition, the influence of publications on epididymitis was primarily evaluated using citation-based metrics. However, citation frequency does not fully equate to research quality or clinical relevance (36). Citation patterns may be shaped by various non-academic factors. For instance, articles published in high-impact journals are more likely to receive citations, and English-language publications generally attract greater international attention than those published in other languages. Nonetheless, because this study employed a multidimensional analytical framework incorporating multiple bibliometric indicators, the impact of these potential biases on the overall trends is expected to be relatively limited.

## 6 Conclusion

Using bibliometric methods and scientific knowledge mapping, this study comprehensively analyzed the developmental trends and research hotspots in epididymitis. The findings reveal an evolutionary trajectory characterized by a transition from clinical diagnosis and treatment to mechanistic exploration, from infectious etiology to immunopathology, and from acute disease processes to chronic pathological changes. This shift reflects the growing emphasis on long-term mechanistic studies rather than short-term clinical management. Currently, key research hotspots in this field include the immunopathological mechanism and immune microenvironment of chronic epididymitis, sexually transmitted pathogens, and epididymitis-induced male infertility. Accordingly, future research should further elucidate the immunopathological mechanisms underlying epididymitis and develop personalized therapeutic strategies targeting its chronic complications, such as chronic pain and obstructive azoospermia. These strategies may include anti-inflammatory and immunomodulatory therapies, antifibrotic agents, and advanced microsurgical techniques, with the ultimate goal of improving patients’ quality of life.

## Data Availability

Publicly available datasets were analyzed in this study from PubMed and Web of Science and are included in the article/supplementary material, further inquiries can be directed to the corresponding author/s.
